# Curcumin Protects against 1-Methyl-4-phenylpyridinium Ion- and Lipopolysaccharide-Induced Cytotoxicities in the Mouse Mesencephalic Astrocyte via Inhibiting the Cytochrome P450 2E1

**DOI:** 10.1155/2013/523484

**Published:** 2013-06-17

**Authors:** Hai-Yan Gui, Rui-Ni Chen, Yan Peng, Jin-Hua Hu, Zhao Mao, Rui Ning, Wei Shang, Wei Liu, Jing Xiong, Gang Hu, Jian Yang

**Affiliations:** Department of Pharmacology, Nanjing Medical University, 140 Hanzhong Road, Jiangsu, Nanjing 210029, China

## Abstract

Curcumin is extracted from the rhizomes of the ginger family plant *Curcuma longa* L., which has a good protection for liver, kidney, and immune system. However, there is little information about its contribution in protection of astrocytes recently. The present study was undertaken to elucidate the protective effect of curcumin, an herbal antioxidant, on 1-methyl-4-phenylpyridinium ion- (MPP^+^-) and lipopolysaccharide- (LPS-) induced cytotoxicities, as well as the underlying mechanisms by using primary mouse mesencephalic astrocytes. The results showed that curcumin protected the mesencephalic astrocytes from MPP^+^- and LPS-induced toxicities along with reducing reactive oxygen species (*P* < 0.05) and maleic dialdehyde (*P* < 0.05) sufficiently. Moreover, curcumin significantly inhibited the cytochrome P450 2E1 (CYP2E1) expression (*P* < 0.01 at mRNA level, *P* < 0.05 at protein level) and its activity (*P* < 0.05) sufficiently induced by MPP^+^ and LPS in the mouse mesencephalic astrocytes. And curcumin as well as diallyl sulphide, a CYP2E1 positive inhibitor, ameliorated MPP^+^- and LPS-induced mouse mesencephalic astrocytes damage. Accordingly, curcumin protects against MPP^+^- and LPS-induced cytotoxicities in the mouse mesencephalic astrocyte via inhibiting the CYP2E1 expression and activity.

## 1. Introduction

Curcumin (1,7-bis[4-hydroxy 3-methoxy phenyl]-1,6-heptadiene-3,5-dione, Cur) is extracted from the rhizomes of the ginger family plant *Curcuma longa* (*Curcuma longa *L.). Studies suggest that Cur has antiinflammatory [1], antiatherosclerosis [2], antitumor [3], antioxidation [4], scavenging-free radical [5], and other pharmacological effects [6]. Animal model data show that dietary Cur is a strong candidate in the prevention or treatment of major disabling age-related neurodegenerative diseases like Alzheimer's disease, Parkinson's disease (PD), and stroke [7]. Several studies in different experimental models of PD strongly support the clinical application of Cur in PD [8, 9]. However, the underlying mechanisms of Cur treatment of PD are limited.

Astrocytes are the most numerous nonneuronal cell-type in central nervous system and make up 50% of human brain volume. They produce neurotrophic factor, remove toxins and debris from the extracellular space, and regulate innate immunity in the central nervous system [10]. In addition, astrocytes are allowed by some specific enzyme systems to metabolize ammonia, glutamate, free radicals, xenobiotics, and metals. Hence, they protect the neurons from the endogenous and exogenous toxicities. On the other hand, evidence also showed that astrocytes play a vital part in the secondary amplification of cell injury in multiple neurodegenerative disorders such as Parkinson's disease. PD is the second most prevalent neurodegenerative disease. The major pathological features are the loss of dopaminergic neurons in substantia nigra compact, the presence of Lewy's bodies, and the significant decrease of dopamine level in the striatum [11, 12]. Although the exact etiology of the PD is unknown, many studies suggest that multiple factors may be involved, such as heredity, environment, oxidative stress, inflammation, and decline in growth factors [13]. Oxidative stress, which results from the imbalance of reactive oxygen species (ROS) generation and clearance, is one of the important pathogenesis of PD [14]. ROS generation may vary considerably depending on cytochrome P450 enzymes (CYPs). Compared with other CYPs, cytochrome P450 2E1 (CYP2E1) exhibits a higher rate of oxidase activity that causes the formation of ROS during the catalytic cycle. The ROS generation is likely to initiate lipid peroxidation and damage cell membranes [15, 16]. In the brain, CYP2E1 is constitutively expressed in hippocampal pyramid neurons, astrocytes, and endothelial cells [17], and CYP2E1 has been found to be inducible and catalytically active in the central nervous system [18–20]. It has been suggested that 1-methyl-4-phenylpyridinium ion (MPP^+^, the ion form of 1-methyl-4-phenyl-1,2,3,6-tetrahydropyridine, MPTP) and lipopolysaccharide (LPS) increased the expression of CYP2E1 and induced oxidative stress in astrocytes [18, 19]. The increasing evidence shows that CYP2E1 is involved in the MPTP-induced mouse model of PD [21, 22]. 

In this study, we demonstrated that Cur had the cytoprotection against MPP^+^- and LPS-induced toxicities in the primary mouse mesencephalic astrocytes. Meanwhile, Cur decreased the increased ROS and maleic dialdehyde (MDA) amount in the primary culture mesencephalic astrocytes exposed to MPP^+^ and LPS. Combination with an increase of CYP2E1 induced by MPP^+^ and LPS in astrocytes [18, 19], we hypothesized that Cur protects against MPP^+^- and LPS-induced mouse mesencephalic astrocyte damage via inhibiting the CYP2E1 expression and activity.

## 2. Materials and Methods 

### 2.1. Animal and Materials

C57BL/6J mice were kept under environmentally controlled conditions (ambient temperature, 22 ± 1°C; humidity, 40%) on a 12-hour light-dark cycle with food and water ad libitum. All the experiments were approved by Institutional Animal Care and Use Committee (IACUC) of Nanjing Medical University. And efforts were made to minimize animal suffering and to reduce the number of animals used for the experiments.

Dulbecco's modified eagle's medium (DMEM) was from Gibco (Gibco, Grand Island, NY, USA). MPP^+^, LPS, and curcumin (98% purity) were purchased from Sigma-Aldrich (St. Louis, MO, USA). Ethyl alcohol (EtOH) was from Sinopharm (Shanghai, China). Antibody to CYP2E1 was obtained from Abcam (Cambridge, UK). SYBR Premix Ex Taq kit was purchased from Biotechnology Limited Company (Takara, Japan). Tryptase and MTT were purchased from AMRESCO (Solon, OH, USA). Fetal bovine serum (FBS) was purchased from Sijiqing (Hangzhou, China). Trizol was from Invitrogen Life (Carlsbad, CA, USA). MLV and RNase inhibitor were purchased from Promega (Madison, WI, USA). 2′,7′-dichlorofluorescein diacetate (DCFH-DA) was purchased from Biyuntian (Shanghai, China). Lactate dehydrogenase (LDH), MDA, H_2_O_2_, and glutathione peroxidase (GSH-Px) diagnostic kits were from Nanjing Jiancheng Bioengineering Company (Nanjing, China), and the other chemicals were all obtained from Sigma unless otherwise stated.

### 2.2. Primary Mesencephalic Astrocyte Culture

Primary mouse mesencephalic astrocyte culture was prepared from midbrain of newborn of C57BL/6J mice as described previously [23] with slight modifications. Briefly, postnatal (P1-P2) mice were killed by rapid decapitation, the meninges were removed, and the midbrain parts were separated. The midbrain parts were dissociated with 0.25% tryptase at 37°C and terminated by DMEM supplemented with 10% FBS and penicillin/streptomycin. After centrifugation at 1500 rpm for 5 min, the cell pellets were resuspended and seeded on polylysine coated flask. The cultures were maintained at 37°C in a humidified 5% CO_2_-95% air atmosphere [24]. Culture medium was replaced 24 h later and then changed every 2-3 days. After reaching a confluent monolayer of glial cells (10–14 days), mesencephalic astrocytes were replated on other polylysine-coated flasks. The tertiary cultures are ~95–98% astrocytes, with only ~2–5% microglial cells as determined by immunocytochemical staining with anti-GFAP antibody as well as double immunofluorescence for GFAP and microglial marker Iba-1 [24]. The mesencephalic astrocytes were seeded on polylysine coated 6-, 12-, 24-, or 96-well plates for corresponding experiments. 

### 2.3. Cell Viability

Cell viability was detected by 3-(4,5-dimethylthiazol-2-yl) 2,5-diphenyltetrazolium bromide (MTT) assay. Mesencephalic astrocytes were seeded in polylysine coated 96-well plates at a density of 1 × 10^4^ cells/well, pretreated with Cur, diallyl sulphide (DAS), or vehicle (DMSO), and then incubated with MPP^+^ (100 *μ*M) and LPS (1 *μ*g/mL) for 48 h. Then 20 *μ*L of 5 mg/mL MTT was added to cells, and cells were incubated at 37°C for another 4 h. The culture medium was discarded, and 100 *μ*L DMSO was used to dissolve the precipitate. The absorbance was measured at 570 nm using an Automated Microplated Reader ELx800 (BioTek). The cytotoxicity assessment was exhibited by cytotoxicity index, which was calculated as previously described [25]. Each treatment was in quadruplicate. And data came from three independent experiments.

### 2.4. Determination of Lactate Dehydrogenase Release

Mesencephalic astrocytes injury (cytotoxicity) was quantitatively assessed by the measurement of lactate dehydrogenase (LDH) leakage. LDH release in culture medium in the presence of DMSO or Cur for 30 min followed by the addition of MPP^+^ and LPS for 48 h was measured using an LDH diagnostic kit according to the manufacturer's instruction. LDH amount was calculated by measuring absorbance at 450 nm. The data were represented as a percentage of LDH release of control group. Each treatment was in quadruplicate. And data came from three independent experiments.

### 2.5. Hoechst 33342 Staining

To quantify apoptotic mesencephalic astrocytes, mesencephalic astrocytic monolayer was stained with Hoechst 33324. Mesencephalic astrocytes were seeded in polylysine coated 24-well plates and pretreated with Cur or vehicle (DMSO) for 30 min and then incubated with MPP^+^ (100 *μ*M) and LPS (1 *μ*g/mL) for 48 h. The cells were fixed with 4% paraformaldehyde for 20 min, washed with PBS, and then incubated with Hoechst33342 (1 *μ*g/mL) for 10 min. After washing with PBS, the morphological features of apoptosis (chromatin condensation) were monitored by fluorescent microscope (Olympus, Japan) (Acquisition software: DP2-BSW). Cells with condensed nuclei or nuclear condensations were scored as apoptotic mesencephalic astrocytes [26], and each treatment was performed in triplicate. 

### 2.6. Measurement of Reactive Oxygen Species Generation

Formation of ROS was evaluated using 2′,7′-dichlorofluorescein diacetate (DCFH-DA), a membrane-permeable probe. After different treatments, mesencephalic astrocytes were loaded with DCFH-DA (50 *μ*M final concentration) in DMEM for 30 min in the dark and fixed by 4% formaldehyde. After rinsing cells with PBS three times, cells were observed using a fluorescent microscope. At least 400 cells from 12 randomly selected fields per dish were counted. Then, the cells were collected to measure the fluorescence intensity at the excitation wavelength of 485 nm and the emission wavelength of 530 nm [27]. Each treatment was performed in triplicate. And data came from three independent experiments.

### 2.7. Determination of Lipid Peroxidation Products

Mesencephalic astrocytes were seeded in polylysine coated 24-well plates at a density of 2 × 10^5^ cells/well. Cells were pretreated with Cur or vehicle (DMSO) for 30 min and then incubated with MPP^+^ (100 *μ*M) and LPS (1 *μ*g/mL) 400 *μ*L for 48 h. Lipid peroxidation products, namely, the amount of MDA formed by the 2-thiobarbituric acid reaction as thiobarbituric acid reactive substances in the supernatant [28], were measured by using a commercial MDA kit. The spectrophotometric absorbance was assessed at 532 nm in accordance with the manufacturer's instructions. The results were expressed as nmol/mL in 2 × 10^5^ cells. Each treatment was in quadruplicate. And data came from three independent experiments.

### 2.8. The H_2_O_2_ ****Production Assay

Mesencephalic astrocytes were seeded in polylysine coated 12-well plates at a density of 5 × 10^6^ cells/well. Cells were pretreated with Cur or vehicle (DMSO) for 30 min and then incubated with MPP^+^ (100 *μ*M) and LPS (1 *μ*g/mL) 800 *μ*L for 48 h. The supernatants were used to measure H_2_O_2_ production using a H_2_O_2_ diagnostic kit, essentially according to the manufacturer's manual. The H_2_O_2_ products were calculated with a calculation formula provided by the manufacturer's instruction. The H_2_O_2_ product was represented as *μ*mol/L in 5 × 10^5^ cells. Each treatment was in quadruplicate. And data came from three independent experiments. 

### 2.9. GSH-Px Activity Assay

Mesencephalic astrocytes were seeded in polylysine coated 12-well plates at a density of 5 × 10^6^ cells/well. Cells were pretreated with Cur or vehicle (DMSO) for 30 min and then incubated with MPP^+^ (100 *μ*M) and LPS (1 *μ*g/mL) for 48 h. The media were discarded. And cells were rinsed with PBS and harvested in 200 *μ*L of PBS. The cell suspension was sonicated by a sonifier, and cell debris was removed by centrifugation at 1,2000 g for 15 min at 4°C [29]. The GSH-Px activity of cells was measured by using a GSH-Px assay kit, essentially according to the manufacturer's manual. The GSH-Px activity of cells was calculated with a calculation formula provided by the manufacturer's instruction. The GSH-Px activity was represented as unit/min/gram protein. Each treatment was in quadruplicate. And data came from three independent experiments.

### 2.10. Immunocytochemistry

Mesencephalic astrocytes were seeded in polylysine coated 24-well plates at a density of 5 × 10^4^ cells/well in growth medium. After pretreated with Cur (3 *μ*M) or DMSO for 30 min, cells were incubated with MPP^+^, LPS, and EtOH for 48 h. For immunocytochemistry, the cells were fixed with 4% paraformaldehyde for 30 min and then permeabilized with 0.02% Triton X-100 containing 0.1% BSA for 1 h. Immune complexes were formed by incubating cells with primary antibody CYP2E1 (1 : 400) overnight at 4°C followed by incubation with anti-rabbit secondary antibody (1 : 800) for 1 h at 37°C and visualized by DAB. Cell morphology was monitored using an inverted light microscope (Olympus, Japan, Acquisition software: DP2-BSW). Negative control staining was performed without the primary antibodies. EtOH treatment was a positive control of CYP2E1 inducer. After immunocytochemical staining, the astrocytes were captured at a magnification of 200x. Three visions from each well were randomly selected in order to analyze immunoreactive productions of CYP2E1 by using Leica Image Processing and Analysis System. Each treatment was performed in triplicate. And data came from three independent experiments.

### 2.11. Quantitative Real-Time Polymerase Chain Reaction

Total RNA was prepared from mesencephalic astrocytes using Trizol reagent according to the manufacturer's instruction. The first-strand cDNA was synthesized using total RNA (2 *μ*g) at 70°C for 5 min, 42°C for 60 min, and 95°C for 10 min by using an oligo (dT) primer, MMLV reverse transcription, RNase inhibitor, and dNTP mix in a total volume of 20 *μ*L. The cDNAs were quantitative. PCR was conducted with SYBR Premix Ex Taq kit (Takara, Japan). 20 *μ*L of PCR mix contained 10 *μ*L of SYBR Green PCR master mix (2×), 1 *μ*L of each primer, 2 *μ*L of cDNA as template, and 6 *μ*L of water. The PCR amplification and quantification were done in an Applied Biosystems 7300 Real-Time PCR System with SDS software (Applied Biosystems, Warrington, UK). After initial denaturation at 95°C for 30 s, amplifications were carried out for 40 cycles at 95°C for 5 s and 60°C for 31 s. The signals from each target gene were normalized based on the signal from the corresponding GAPDH. PCR primers used in this study were as follows: (1) GAPDH: Forward, 5′-GTATGTCGTGGAGTCTACTGGTGTC-3′, Reverse, 5′-GGTGCAGGATGCATTGCTGACATTC-3′; (2) CYP2E1: Forward, 5′-AAGCGCTTCGGGCCAG-3′, Reverse, 5′-TAGCCATGCAGGA   CCACGA-3′. Each treatment was performed in triplicate. And data came from three independent experiments.

### 2.12. Cytochrome P450 2E1 (CYP2E1) Activity

CYP2E1 activity was measured based on the rate of oxidation of p-nitrophenol (PNP) to p-nitrocatechol in the presence of NADPH by modification of the methods of Reinke and Moyer [30] and Koop [31]. Briefly, after treatment, cells were rinsed with PBS and harvested in 50 *μ*L of 100 mM potassium phosphate buffer, pH 7.4. The cell suspension was sonicated by a sonifier (Nanjing, China), and the cell debris was removed by centrifugation at 12,000 g for 15 min at 4°C. The protein concentration of the samples was estimated with the BCA kit. Standards and blanks were assessed simultaneously. The activity was determined in a final volume of 100 *μ*L containing 100 *μ*g of protein, 100 mM of potassium phosphate, pH 7.4, 0.1 mM of PNP, 1 mM of NADPH and 5 mM of MgCl_2_ followed by incubation at 37°C for 10 min. The reaction was terminated by the addition of 20 *μ*L of chilled trichloroacetic acid (20%, w/v) and centrifuged at 10,000 ×g for 5 min. After being centrifuged, 100 *μ*L of the supernatant was added to 10 *μ*L 10 N of sodium hydroxide. 4-Nitroatechol formation was then spectrophotometrically determined at 510 nm. A standard curve with 4-nitrocatehol was used to calculate CYP2E1 activity. The results were expressed as nmol per minute per mg of protein. Each treatment was in quadruplicate. Several controls were performed including incubation protein or regeneration system. And data came from three independent experiments.

### 2.13. Statistical Analysis

All data were presented as the mean ± SD (standard deviation). Statistical analysis between control group and Cur treatment group was performed with two-tailed independent Student's *t*-tests, using SPSS 10.0 for Windows (SPSS, Inc., Chicago, IL, USA). And statistical analysis for multiple comparisons was performed by a one-way ANOVA test with Turkey's corrections. Differences were considered statistically significant when the *P* value was less than 0.05, 0.01, or 0.001.

## 3. Results and Discussion

### 3.1. Results

#### 3.1.1. Cur at Low Concentration Had No Cytotoxicity to the Mouse Mesencephalic Astrocytes

**Figure 1:**
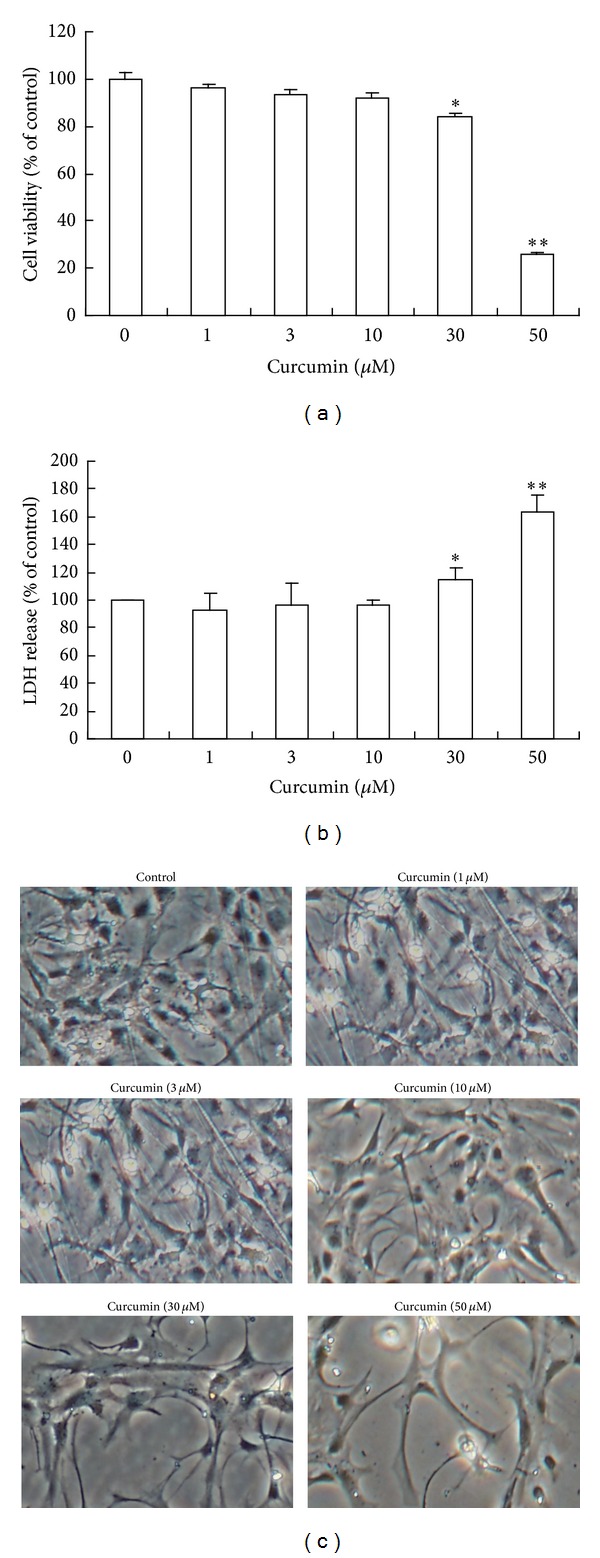
Effects of Cur on the cytotoxicity of the primary cultured mesencephalic astrocytes. (a) Cell viability of the primary cultured mesencephalic astrocytes. (b) LDH release of the primary cultured mesencephalic astrocytes. (c) Morphologies of the primary cultured mesencephalic astrocytes. Primary cultured mesencephalic astrocytes were treated with the indicated concentrations of Cur (1, 3, 10, 30, and 50) (*μ*M) for 48 h. The cell viability was determined with MTT. LDH release was measured by the LDH diagnostic kit. Morphological analysis was taken under bright field (magnification 40x). And data are presented as mean ± SD of three independent experiments, *P < 0.05  and **P < 0.01 compared to the control group.

As shown in Figure 1(a), the viability of the mesencephalic astrocytes was unchanged when cells were treated with Cur from 1 *μ*M to 10 *μ*M compared to that of the control. However, a significant decrease of the mesencephalic astrocytes viability was observed when cells were treated with 30 *μ*M and 50 *μ*M of Cur. LDH release in culture medium was employed to indicate cell membrane damage. Similar to MTT assay (Figure 1(b)), Cur at low concentrations (1 *μ*M−10 *μ*M) did not change the LDH release. But Cur at high concentrations (30 *μ*M and 50 *μ*M) significantly increased the LDH release in the mesencephalic astrocytes. Consistently, mesencephalic astrocytes, which were treated with low concentrations (1 *μ*M, 3 *μ*M, and 10 *μ*M) of Cur, possessed mainly rounded or oval body and exhibited extensive processes. The body and processes of mesencephalic astrocytes were near normalcy (control). In contrast, the body of mesencephalic astrocytes which were treated with high concentrations (30 *μ*M, 50 *μ*M) of Cur became more asperous with shrunken morphologies, and processes of mesencephalic astrocytes were reduced in size and decreased in number (Figure 1(c)). 

The data suggested that Cur at low concentrations had no cytotoxicity but at high concentrations had some cytotoxicities in the mouse mesencephalic astrocytes. So we chose the low concentrations of Cur (1 *μ*M, 3 *μ*M, and 10 *μ*M) for subsequent experiments.

#### 3.1.2. Cur Ameliorated the Cell Damage Induced by MPP^+^ and LPS in the Mouse Mesencephalic Astrocytes

**Figure 2:**
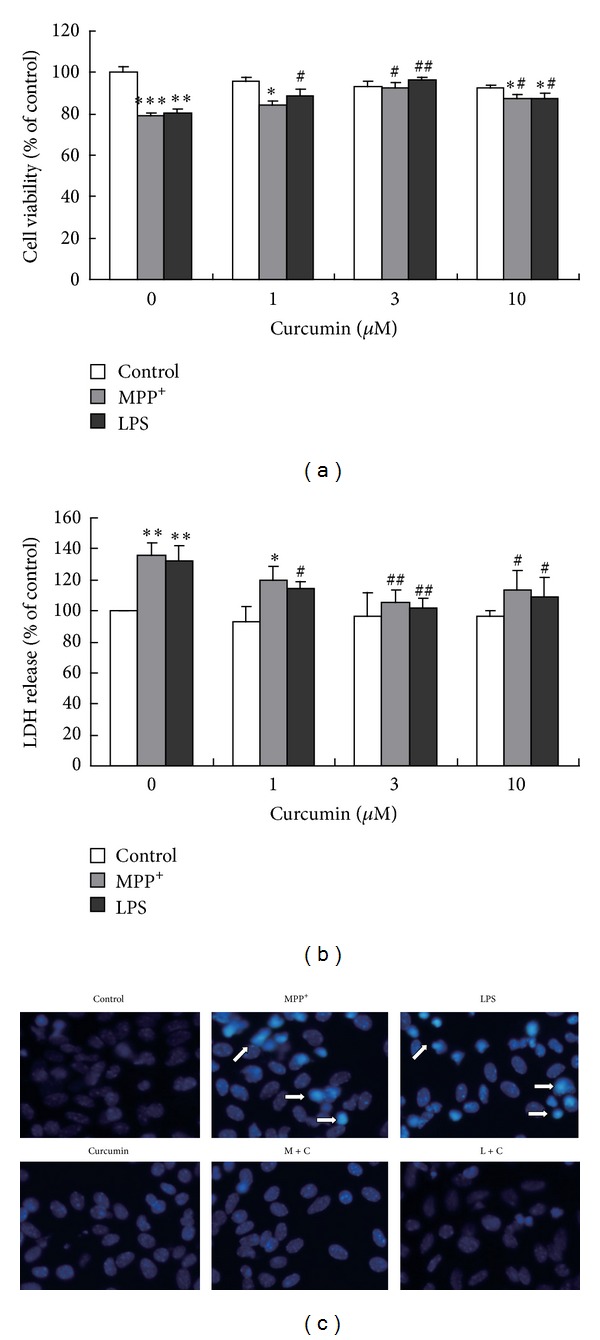
Cur protected the mesencephalic astrocytes from cytotoxicity induced by MPP^+^ and LPS. (a) Cell viability of the mesencephalic astrocytes. (b) LDH release of the mesencephalic astrocytes. (c) Apoptosis of the mesencephalic astrocytes. The primary cultured mesencephalic astrocytes were nonpretreated or pretreated with vehicle (DMSO) or Cur (1, 3, and 10) (*μ*M) for 30 min and then incubated with PBS, MPP^+^ (100 *μ*M), or LPS (1 *μ*g/mL) for 48 h. The cell viability was determined with MTT. LDH release was measured by the LDH diagnostic kit. Nuclear condensation was assessed by staining with Hoechst33342 (magnification 200x). Data are presented as mean ± SD of three independent experiments, *P < 0.05,  **P < 0.01, and ***P < 0.001 compared to the control group; ^#^ *P* < 0.05, and ^##^ *P* < 0.01 compared to the MPP^+^ or LPS treatment alone group.

It has been reported that exposure to MPP^+^ and LPS potentially results in astrocytes damage or death [24]. We therefore investigated whether Cur protected against MPP^+^ and LPS in the mesencephalic astrocytes. The results showed that Cur (1 *μ*M, 3 *μ*M, and 10 *μ*M) attenuated the reduction of cell viability and the increase of LDH release induced by MPP^+^ and LPS in the primary cultured mouse mesencephalic astrocytes. The most significant protective effect of Cur was at 3 *μ*M (Figures 2(a) and 2(b)). Therefore, 3 *μ*M of Cur was chosen to explore the mechanism in the following experiments. 

The nuclear staining assay was used to assess the morphological changes of apoptosis in the mesencephalic astrocytes. The normal cells exhibited uniformly dispersed chromatin and intact cell membrane. The impaired cells appeared typical characteristics of apoptosis, including apoptotic nuclear condensation. Similar to the results of cell viability and LDH release, Cur attenuated the mesencephalic astrocytes apoptosis induced by MPP^+^ and LPS (Figure 2(c)). 

The data suggested that Cur attenuated the mouse mesencephalic astrocyte damage induced by MPP^+^ and LPS (Figures 2(a), 2(b), and 2(c)).

#### 3.1.3. Cur Decreased the ROS and MDA Production Induced by MPP^+^ and LPS in the Mouse Mesencephalic Astrocytes

**Figure 3:**
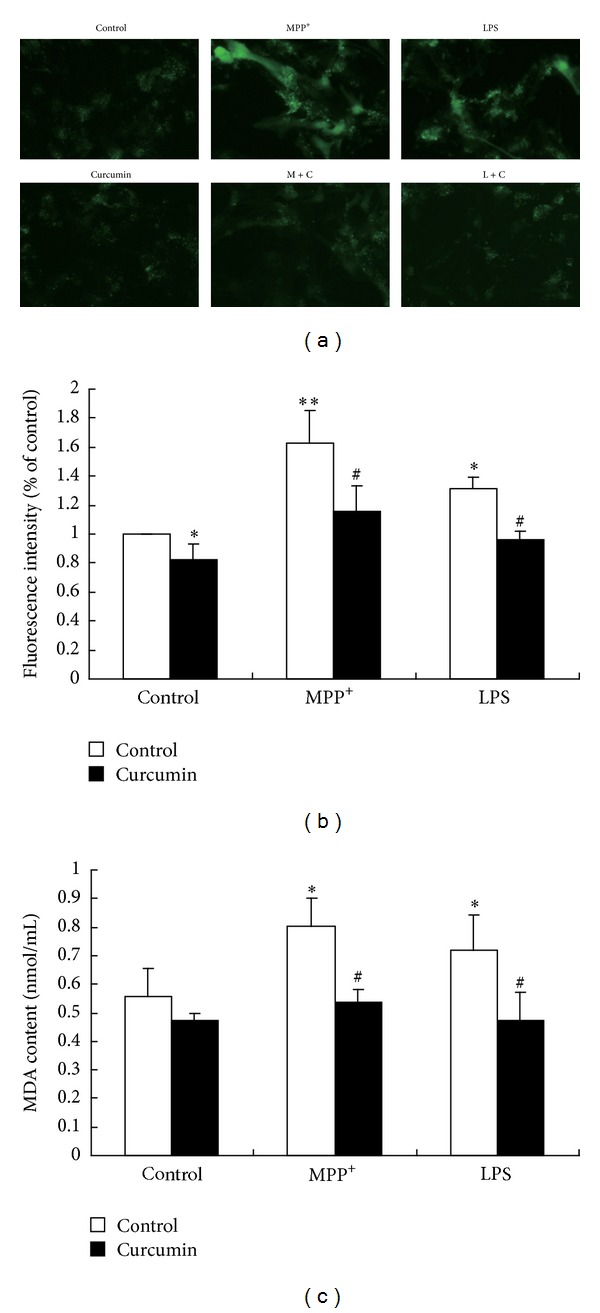
Cur decreased ROS and MDA productions in primary mesencephalic astrocytes. (a) DCFH-DA fluorescence (green) images of the primary cultured mesencephalic astrocytes. (b) Quantification of DCFH-DA fluorescence intensity. (c) MDA assay in medium of the mesencephalic astrocytes. The primary cultured mesencephalic astrocytes were nonpretreated or pretreated with vehicle (DMSO) or Cur (3 *μ*M) for 30 min and then incubated with PBS, MPP^+^, or LPS for 48 h. The cells were loaded with DCFH-DA (50 *μ*M final concentration) in DMEM for 30 min in the dark and fixed by 4% formaldehyde. After rinsing cells with PBS three times, cells were observed using a fluorescent microscope (magnification 200x). MDA amount was measured by using the MDA diagnostic kit. Data are presented as mean ± SD of three independent experiments, *P < 0.05  and **P < 0.01 compared to the control group; ^#^ *P* < 0.05 compared to the MPP^+^ or LPS treatment alone group.

Since ROS played an important role in cell death, we next investigated the intracellular ROS formation using a fluorescent sensitive probe (DCFH-DA). As shown in Figures 3(a) and 3(b), when the mesencephalic astrocytes were treated with MPP^+^ and LPS for 48 h, the intracellular ROS level significantly increased compared with that of the control, respectively. The results revealed that MPP^+^ and LPS enhanced ROS generation in the primary mouse mesencephalic astrocytes. Cotreatment with 3 *μ*M of Cur reduced the intracellular ROS production and significantly attenuated an increase of ROS caused by MPP^+^ and LPS in the primary mouse mesencephalic astrocytes (Figures 3(a) and 3(b)).

ROS can initiate lipid peroxidation; MDA is one of the most frequently used indicators of lipid peroxidation. When the mesencephalic astrocytes were exposed to MPP^+^ and LPS, the MDA amount in culture medium increased significantly. Being cotreated with Cur, the amount of MDA decreased markedly in the mesencephalic astrocytes (Figure 3(c)). These data suggested that Cur decreased the increased ROS and MDA amounts induced by MPP^+^ and LPS in the primary mouse mesencephalic astrocytes.

#### 3.1.4. Cur and DAS Ameliorated Cell Viability of the Primary Mesencephalic Astrocytes Induced by MPP^+^ and LPS

**Figure 4:**
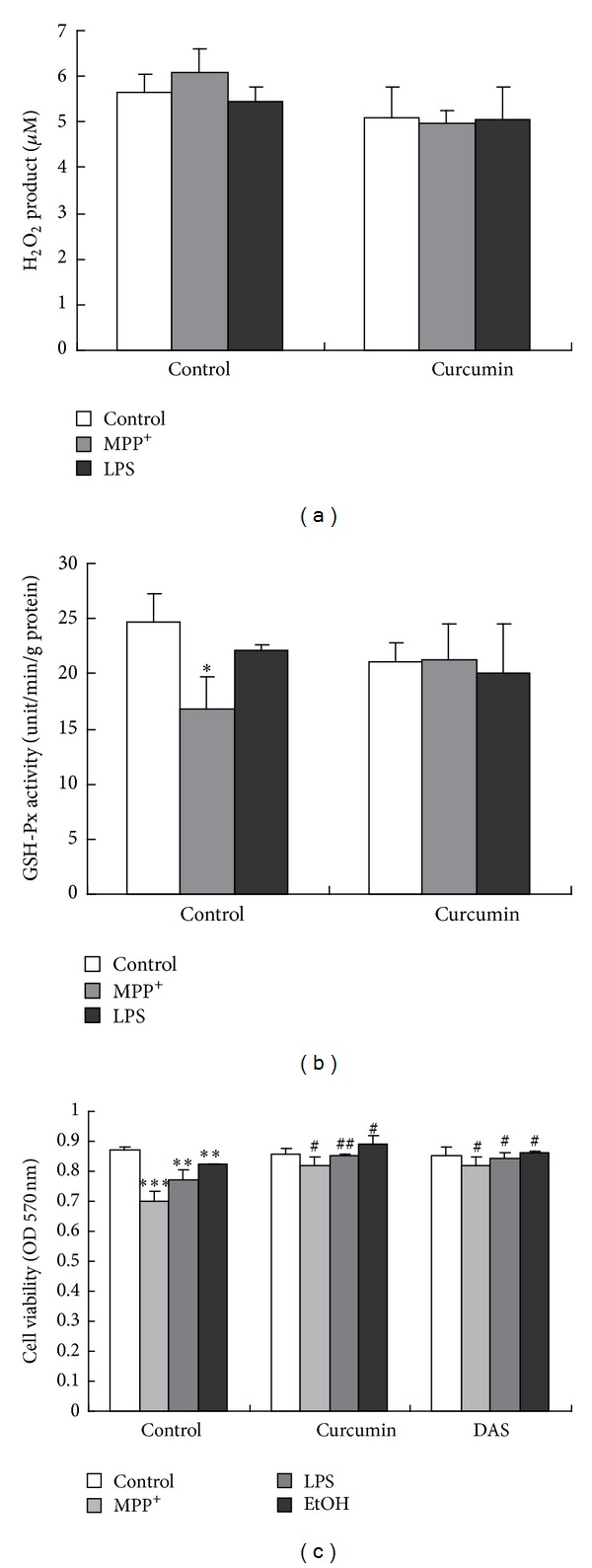
Cur as well as DAS ameliorated cell viability of the primary mesencephalic astrocytes induced by MPP^+^ and LPS. (a) The H_2_O_2_ production of the primary cultured mesencephalic astrocytes. (b) The GSH-Px activity of the primary cultured mesencephalic astrocytes. The primary cultured mesencephalic astrocytes were nonpretreated or pretreated with vehicle (DMSO) and Cur (3 *μ*M) for 30 min and then incubated with PBS, MPP^+^, or LPS for 48 h. The cultured media were measured for H_2_O_2_ production with H_2_O_2_ assay kit. And the lysates determined GSH-Px activity using GSH-Px assay kit. (c) Effect of Cur and DAS on the cell viability of the primary cultured mesencephalic astrocytes induced by MPP^+^ and LPS. The primary cultured mesencephalic astrocytes were nonpretreated or pretreated with vehicle (DMSO), Cur (3 *μ*M), and DAS (500 *μ*M) and then incubated with PBS, MPP^+^, LPS, or EtOH for 48 h. EtOH was as a positive CYP2E1 inducer. Data are presented as mean ± SD of three independent experiments, *P < 0.05,  **P < 0.01, and  ***P < 0.001 compared to the control group; ^#^ *P* < 0.05 and  ^##^ *P* < 0.01 compared to the MPP^+^ or LPS treatment alone group.

It was reported that curcumin generated H_2_O_2_ in astrocytes [32]. In order to explore the role of Cur in protecting the astrocytes, we next examined the H_2_O_2_ product and GSH-Px activity in the mouse mesencephalic astrocytes treated with Cur alone or combination with MPP^+^ and LPS. As shown in Figure 4, the H_2_O_2_ products did not significantly change in the mouse mesencephalic astroytes after being exposed to MPP^+^ and LPS, as well as to Cur (Figure 4(a)). The GSH-Px activity significantly decreased in the mouse mesencephalic astroytes induced by MPP^+^, not by LPS. Cur attenuated the decrease of GSH-Px activity in the mouse mesencephalic astroytes induced by MPP^+^ (Figure 4(b)). Both Cur and DAS (a CYP2E1 positive inhibitor) significantly ameliorated cell viability of the mouse mesencephalic astroytes after being exposed to MPP^+^, LPS, and EthOH (Figure 4(c)).

The data implied that the effect of Cur on the decrease of ROS and MDA in the primary mouse mesencephalic astrocytes induced by MPP^+^ and LPS was not caused by promoting H_2_O_2_ production. Cur as well as DAS protected the mouse mesencephalic astroytes from MPP^+^- and LPS-induced cytotoxicities.

#### 3.1.5. Cur Inhibited Both CYP2E1 Expression and Activity in the Mouse Mesencephalic Astrocytes Induced by MPP^+^ and LPS

**Figure 5:**
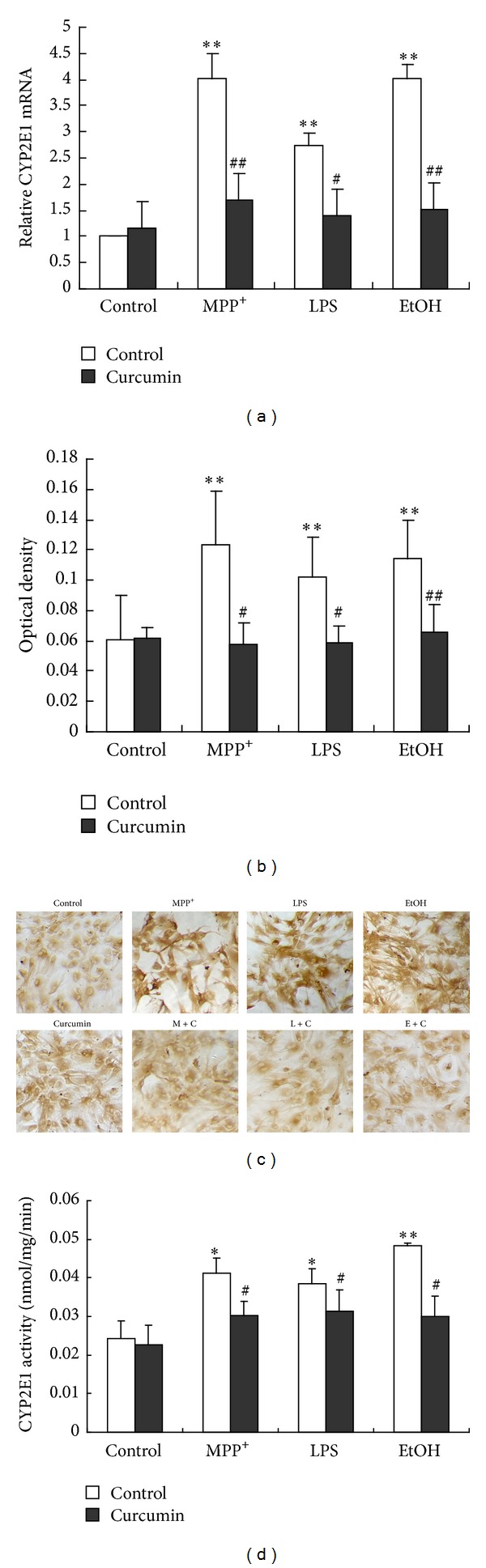
Cur inhibited the CYP2E1 expression and activity in the primary mesencephalic astrocytes. (a) Effect of Cur on CYP2E1 mRNA level. (b)-(c) Effect of Cur on CYP2E1 protein level (immunocytochemistry images and the optical density). (d) Effect of Cur on CYP2E1 activity. Primary cultured mesencephalic astrocytes were nonpretreated or pretreated with DMSO or Cur (3 *μ*M) for 30 min and then incubated with PBS, MPP^+^, LPS, or EtOH for 48 h. EtOH was a positive CYP2E1 inducer. CYP2E1 mRNA level was determined with real-time PCR. CYP2E1 protein level was determined with immunocytochemistry described in Section 2 (magnification 200x). And CYP2E1 activity was measured by hydroxylation rate of p-nitrophenol. Data are presented as mean ± SD of three independent experiments, *P < 0.05,  **P < 0.01, compared to the control group;  ^#^ *P* < 0.05,  ^##^ *P* < 0.01 compared to the MPP^+^, LPS, or EtOH treatment alone group.

Our previous work showed that CYP2E1 was an important responsibility for MPP^+^ and LPS cytotoxicities [24]. A CYP2E1 inhibitor protected the mouse astroytes from MPP^+^- and LPS-induced cytotoxicity [24]. Presumably, the mechanism of Cur neuroprotection was probably through inhibiting CYP2E1 expression and activity in the mesencephalic astrocytes. In order to test this probability, we further investigated the effects of Cur on the CYP2E1 expression and activity in the mesencephalic astrocytes induced by MPP^+^, LPS and ethyl alcohol (EtOH). EtOH was a positive control of CYP2E1 inducer in this experiment. The results showed that when the mesencephalic astrocytes were exposed to MPP^+^, LPS, and EtOH, the CYP2E1 mRNA increased by 325%, 222%, and 292%, respectively. Furthermore, Cur almost abolished the increase of CYP2E1 expression induced by MPP^+^, LPS, and EtOH at mRNA level (Figure 5(a)). Similarly, the CYP2E1 immunostaining was much stronger in the mouse mesencephalic astrocytes after exposure to MPP^+^, LPS, and EtOH (Figure 5(c)). Semiquantitative analysis showed that the mean optical density of CYP2E1 increased to 187%, 161%, and 175%, respectively, and Cur almost abolished the increase of CYP2E1 expression induced by MPP^+^, LPS, and EtOH at protein level (Figure 5(b)). Since the expression of CYP2E1 was induced by MPP^+^, LPS, and EtOH in the mouse mesencephalic astrocytes, what happened to the activity of CYP2E1 in MPP^+^, LPS, and EtOH-exposed mesencephalic astrocytes? We measured the CYP2E1 activity by the hydroxylation of p-nitrophenol to 4-nitrocatechol. The results showed that the CYP2E1 activity of the mouse mesencephalic astrocytes increased by 1.69-fold, 1.58-fold, and 1.83-fold after exposing to MPP^+^, LPS, and EtOH, respectively, (Figure 5(d)). Cur substantially inhibited both the increase of CYP2E1 expression and activity in the mouse mesencephalic astrocytes induced by MPP^+^, LPS, and EtOH. However, Cur treatment alone seemed to have little effect on the constitutive CYP2E1 expression and activity.

The data implied that Cur, as well as DAS, inhibited both CYP2E1 expression and activity in the mouse mesencephalic astroytes induced by MPP^+^, LPS, and EtOH.

### 3.2. Discussion

At present, if not all, most of the disabling clinical abnormalities of PD are attributed to a profound decrease in brain dopamine content in the striatum, which is induced by the remarkable loss of dopaminergic neurons in the substantia nigra pars compacta (SNpc) [12]. Therefore, the most potent treatment for PD remains the administration of levodopa (L-dopa), a precursor of dopamine, to alleviate PD symptoms. However, the chronic administration of L-dopa often produces motor and psychiatric side effects, which may limit L-dopa use [31]. So it is expected to find the natural compounds with the protective effect and no or less side effects for treatment of PD.

Recent studies have proven that Cur is a strong candidate in the prevention or treatment of major disabling age-related neurodegenerative diseases like Alzheimer's disease, PD, and stroke [7, 8, 33]. Cur can effectively protect against ethanol-induced liver damage [34] and ethanol-induced central nervous system neurodegeneration *in vivo* [35]. It can directly protect the SHSY-5Y cells from MPP^+^ toxicity [33]. Low doses of Cur can decrease the 6-hydroxydopamine- and dopamine-induced neurotoxicities *in vivo* [36, 37]. Current studies show that Cur protects SH-SY5Y and PC12 cells from *α*-synuclein-induced cytotoxicities through antioxidation and scavenging-free radical [38, 39]. However, there is little information about what are the effects of Cur on the mesencephalic astrocytes which maintain homeostasis and protect against the endogenous and exogenous toxicities in the central nervous system. In the present study, we investigated the effects of Cur on the primary mouse mesencephalic astrocytes which play a crucial role in PD. We demonstrated that Cur significantly protected against MPP^+^- and LPS-induced mouse mesencephalic astrocytes damage at low concentration (1 *μ*M−10 *μ*M). In contrast, high concentrations of Cur (30 *μ*M, 50 *μ*M) were cytotoxicity to the mesencephalic astrocytes (Figures 1(a), 1(b), and 1(c)). Therefore, it may not be a simple stoichiometric reaction and the function of Cur was biphasic: Cur had nontoxicity and protective effect on the mesencephalic astrocytes at low concentrations (no more than 30 *μ*M), while at high concentrations (more than 30 *μ*M), Cur promoted the mesencephalic astrocytes damage. The reason was probably that Cur had multiple actions, such as antioxidation [4], scavenging-free radical [5], and inducing apoptosis [40]. 

**Figure 6:**
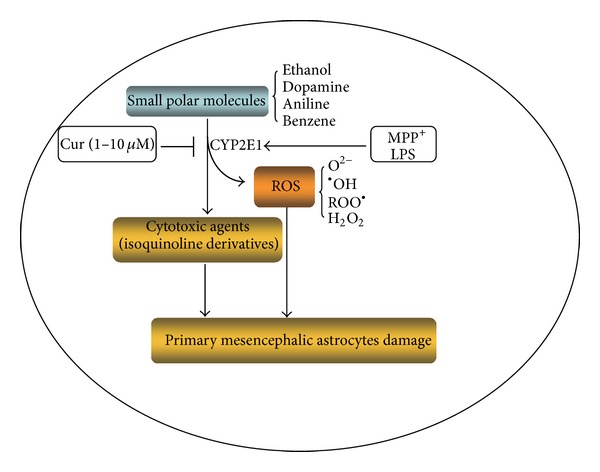
The schematic mechanism of Cur in protecting against MPP^+^- and LPS-induced cytotoxicities in the primary mesencephalic astrocytes. CYP2E1 is a main enzyme catalyzing the small polar molecules into oxgen ions, peroxides, and other cytotoxic agents such as isoquinoline derivatives. MPP^+^ and LPS activated the CYP2E1 and induced mesencephalic astrocyte damage. Cur inhibited the CYP2E1 expression and activity, reduced the ROS production, and protected against MPP^+^- and LPS-induced cytotoxicities in the mesencephalic astrocytes.

Oxidative stress plays an important role in PD, and dopamine-rich areas of the brain, such as mesencephalon, are particularly vulnerable to oxidative stress, because metabolism of dopamine itself leads to enhancement of ROS generation [41]. Studies showed that Cur served as a chelator and directly bound to Fe^2+^, which catalyzes formation of free radicals via the Fenton reactions [42]. Our results also showed that Cur attenuated the three toxins-induced increases of ROS and MDA (lipid peroxidation production). It has reported that Cur may terminate lipid peroxidation by induction of enzymatic and nonenzymatic antioxidants [43]. Accordingly, the reduction of ROS and MDA afforded by Cur in the mouse mesencephalic astrocytes induced by MPP^+^, LPS is likely attributable to its antioxidant effects. Recent study reported that Cur generated H_2_O_2_ in astrocytes [32]. However, our study showed that Cur did not significantly promote the H_2_O_2_ generation in the mouse mesencephalic astrocytes. The possible reason was that the concentration of Cur that we used was much lower (3 *μ*M) than that in report [32]. CYP2E1 is likely to be involved in the metabolism of the endogenous and exogenous compounds, which can cause neurodegenerative diseases such as PD [44]. In addition, CYP2E1 exhibits a high rate of oxidase activity that causes the formation of ROS during its catalytic cycle [16]. The CYP2E1 activation increases ROS production in astrocytes [24]. In this study, we found that Cur attenuated the primary cultured mouse mesencephalic astrocytes damage and apoptosis along with the decrease of ROS and MDA production induced by MPP^+^ and LPS. Moreover, Cur as well as DAS, a CYP2E1 positive inhibitor [45], ameliorated the cell viability in the mesencephalic astrocytes induced by MPP^+^ and LPS. These data implied that the inhibition of CYP2E1 was involved in the protection of Cur in the mesencephalic astrocytes. Therefore, in the next step, we investigate the effects of Cur on CYP2E1 expression and activity in mesencephalic astrocytes after being exposed to MPP^+^ and LPS. Consistently, MPP^+^, LPS, and EtOH virtually triggered the CYP2E1 expression and enzymatic activity in mesencephalic astrocytes. Here, EtOH was a positive control of CYP2E1 inducer in this experiment. And Cur significantly lowered the increase of the CYP2E1 expression and activity in mesencephalic astrocytes induced by MPP^+^, LPS, and EtOH. Our previous work showed that the inhibition of CYP2E1 by DAS completely protected astrocytes from MPP^+^, LPS, and EtOH-induced oxidative stress [24]. It was also found that Cur as well as DAS ameliorated the cell viability of the mesencephalic astrocytes induced by MPP^+^, LPS, and EtOH in this study. These data imply that Cur protected the mesencephalic astrocytes from MPP^+^ and LPS. And the potential mechanism was that Cur inhibited CYP2E1 expression and activity and decreased the ROS generation subsequently in the mesencephalic astrocytes (Figure 6). It was reported Cur alleviated ethanol-induced hepatocytes oxidative damage involving heme oxygenase-1 (HO-1) induction [46]. Cur also potently induced HO-1 expression and activity in rat astrocytes [47]. HO-1 was the rate-limiting enzyme decomposing heme into biliverdin, free iron, and carbon monoxide (CO) by adding an oxygen molecule to the porphyrin ring of heme. CO inhibited ethanol-induced CYP2E1 activity and hepatotoxicity but had no influence on CYP2E1 protein expression [48]. Together, Cur might protect against MPP^+^- and LPS-induced toxicities in the mesencephalic astrocytes not only by increasing the HO-1 activity but also by inhibiting CYP2E1 expression and activity. 

## 4. Conclusion

Our work points to several important conclusions. First, we report for the first time that Cur can protect against MPP^+^- and LPS-induced cytotoxicities in the primary mouse mesencephalic astrocytes. And inhibition of both CYP2E1 expression and activity is suggested as the mechanism of protective effects of Cur against MPP^+^- and LPS-induced injuries in the primary mouse mesencephalic astrocytes. Second, Cur decreases the CYP2E1 expression and activity in the primary mouse mesencephalic astrocytes, indicating that Cur affects metabolism of the endogenous and exogenous compounds in the central nervous system. Taken together, this study provides evidence to explore the possible use of Cur, a very low toxic natural compound as a therapeutic approach in PD at low concentration, and provides new insight into understanding the role of Cur in the drug metabolism enzymes in the central nervous system.

## Abbreviations

Cur: Curcumin
ROS: Reactive oxygen species
PD: Parkinson's disease
CYPs: Cytochrome P450 enzymes
CYP2E1: Cytochrome P450 2E1
MPP^+^: 1-Methyl-4-phenylpyridinium ion
LPS: Lipopolysaccharide
MDA: Maleic dialdehyde
H_2_O_2_: Hydrogen peroxide
GSH-Px: Glutathione peroxidase
DMEM: Dulbecco's modified Eagle's medium
MTT: 3-(4,5-Dimethylthiazol-2-yl) 2,5-Diphenyltetrazolium bromide
LDH: Lactate dehydrogenase
DAB: Diaminobenzidine
DAS: Diallylsulphide
EtOH: Ethyl alcohol
DCFH-DA: 2′,7′-Dichlorofluorescein diacetate
FBS: Fetal bovine serum
DMSO: Dimethyl sulfoxide
L-dopa: Levodopa.
